# Supplementary material for: Optical tweezers-controlled hotspot for sensitive and reproducible surface-enhanced Raman spectroscopy characterization of native protein structures
Source: Nat Commun. 2021 Feb 26;12:1292. doi: 10.1038/s41467-021-21543-3 (PMC7910584; doi:10.1038/s41467-021-21543-3)
Supplement: Supplementary file 1 — Supplementary Information [file 41467_2021_21543_MOESM1_ESM.pdf]

Supplementary Information

Optical tweezers-controlled hotspot for sensitive and reproducible surface-enhanced Raman spectroscopy characterization of native protein structures

Xin Dai[1, 2], Wenhao Fu[1], Huanyu Chi[1], Vince St. Dollente Mesias[1], Hongni Zhu[1], Cheuk Wai Leung[1], Wei Liu[3, *], and Jinqing Huang[1, *]

[1]Department of Chemistry, The Hong Kong University of Science and Technology, Clear Water Bay, Hong Kong, China.

[2]Laboratory for Synthetic Chemistry and Chemical Biology, Health@InnoHK, Hong Kong Science Park, Hong Kong, China

[3]State Key Laboratory of Synthetic Chemistry, Department of Chemistry, The University of Hong Kong, Pokfulam Road, Hong Kong, China

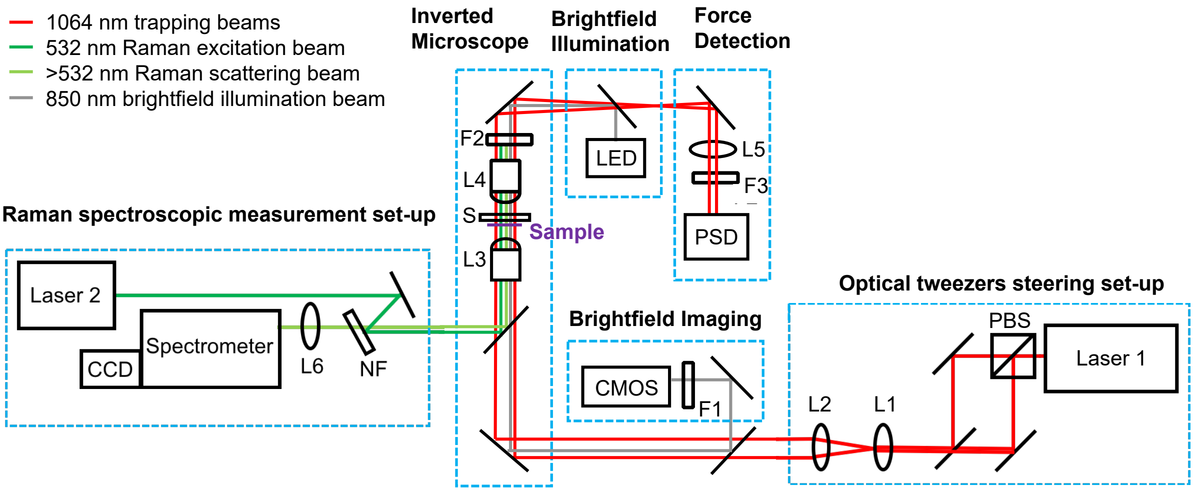

Supplementary Figure 1. Instrumental layout for the optical tweezers coupled Raman spectroscopic experimental platform. Abbreviations: Laser 1, 1064 nm Ytterbium linearly polarized CW laser (IPG Photonics); PBS, polarizing beamsplitter cube; L1, L2, lens; F1, filter; CMOS, complementary metal oxide semiconductor camera; L3, objective (Nikon, 60X 1.2 N.A.); S, sample holder on two-axis motorized translation micro-stage; L4, condenser (Leica, 60X 1.4 N. A.); LED, 850 nm infrared light emitting diode; L5, lens; F2, F3, filter; PSD, position sensitive detector; Laser 2, 532 nm fiber laser (CNI); NF, notch filter (Thorlab); L6, lens; Spectrometer, IsoPlane SCT-320, 1200 lines/mm (Princeton Instrument); CCD, liquid nitrogen-cooled charge-coupled device camera (Princeton Instrument).

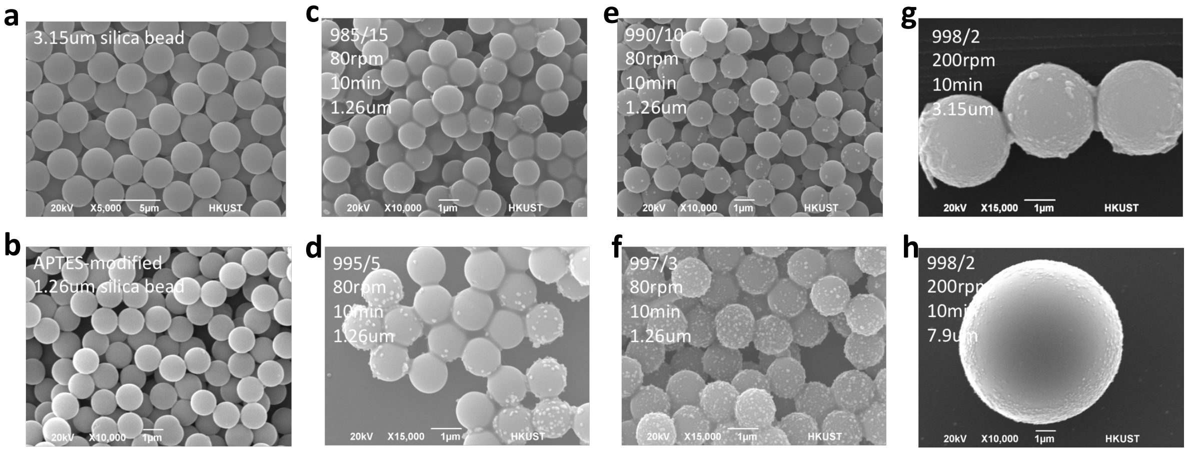

Supplementary Figure 2. SEM images of the silica beads and AgNP-coated beads prepared under varying fabrication parameters. The scale bar is shown in each image. All micrographs are representative images of three independent measurements.

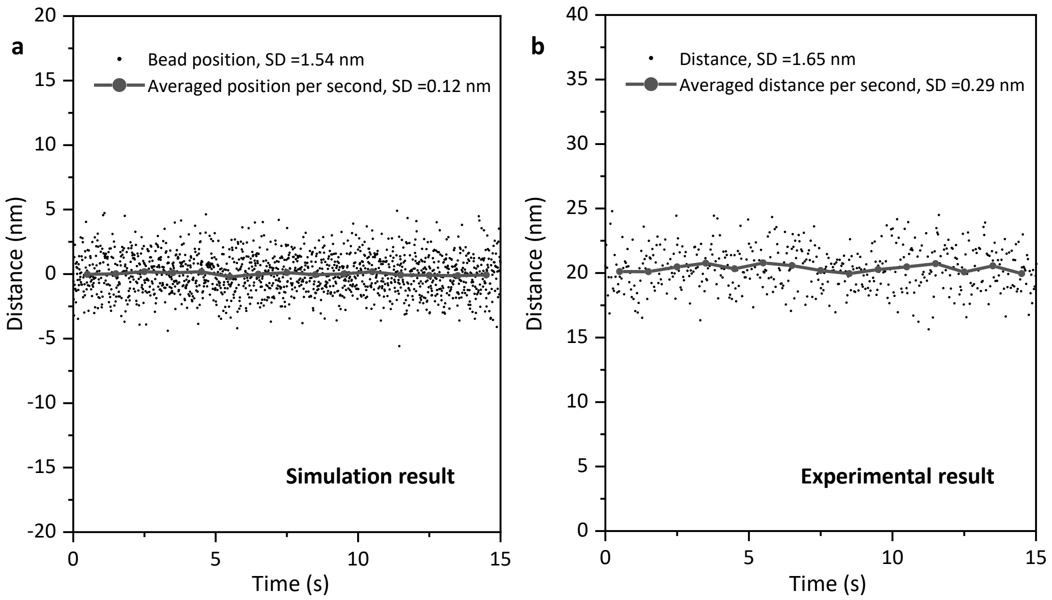

Supplementary Figure 3. **a**: The theoretical simulation of the Brownian fluctuation of one trapped bead (R=0.63 μm, k=2.0 pN/nm, sampling rate $\Delta t=10^{-2}$ s), giving the SD=0.12 nm for the average bead positions in 1 second over 15 seconds. **b**: The experimental time traces of the distance between the two AgNP-coated beads trapped at 20 nm at the sampling rate of $\Delta t=3 \times 10^{-2}$ s, demonstrating the SD=0.29 nm for the average bead distance in 1 second over 15 seconds.

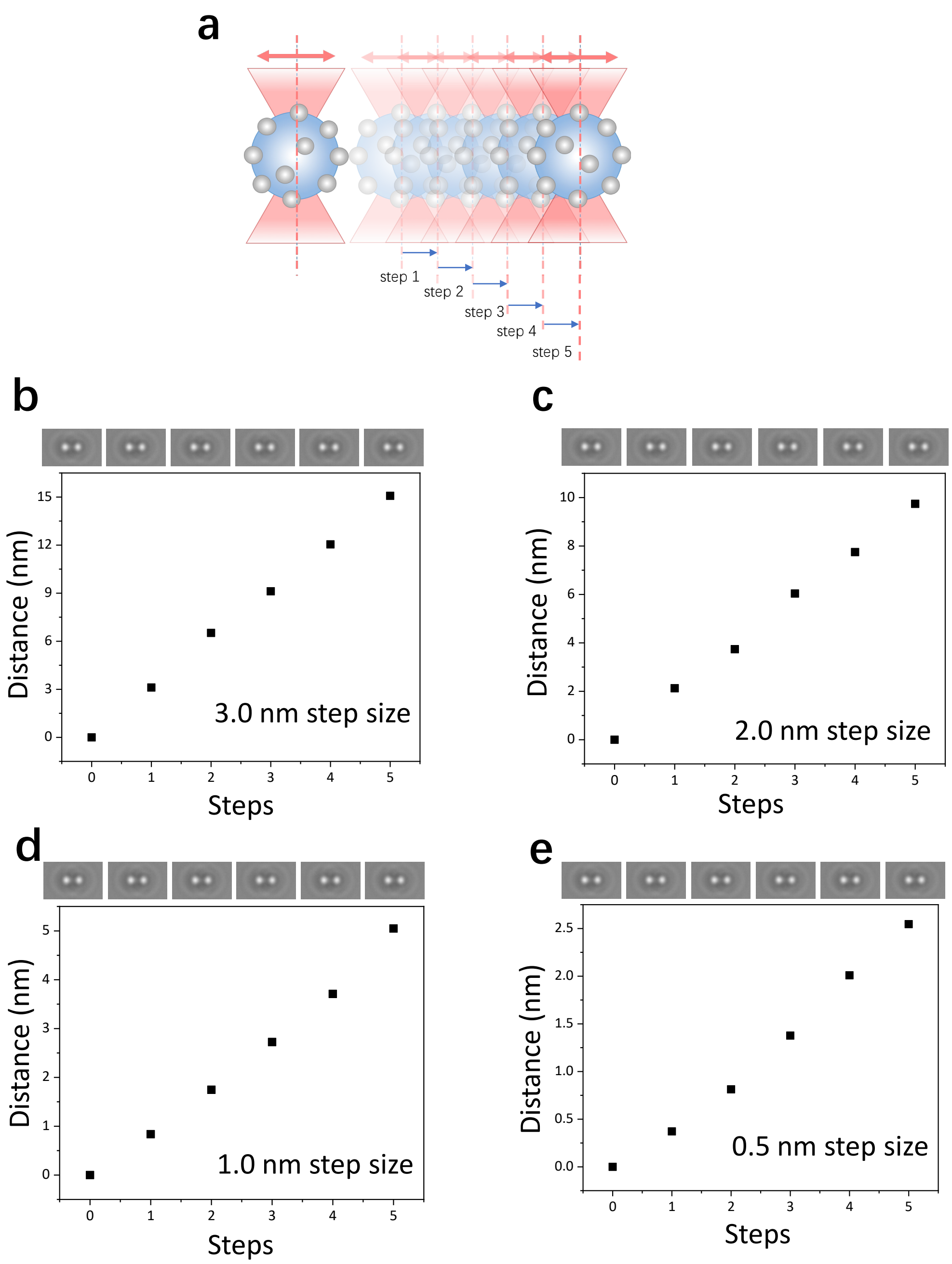

Supplementary Figure 4. Incremental movements of two AgNP-coated beads by optical tweezers at different step sizes. **a**: Illustration of the incremental bead movements. One bead (on the left side) was kept stationary while the other bead (on the right side) was moved away in the incremental steps (5 steps in total). After each steering operation, the distance data points of the two trapped AgNP-coated beads within 1 second was averaged and plotted as a function of the step number with the real-time camera images in **b**: at 3.0 nm step size, **c**: at 2.0 nm step size, **d**: at 1.0 nm step size, and **e**: at 0.5 nm step size, respectively, demonstrating the sub-nm spatial resolution in the bead trapping and the gap formation on our optical tweezers-coupled Raman platform.

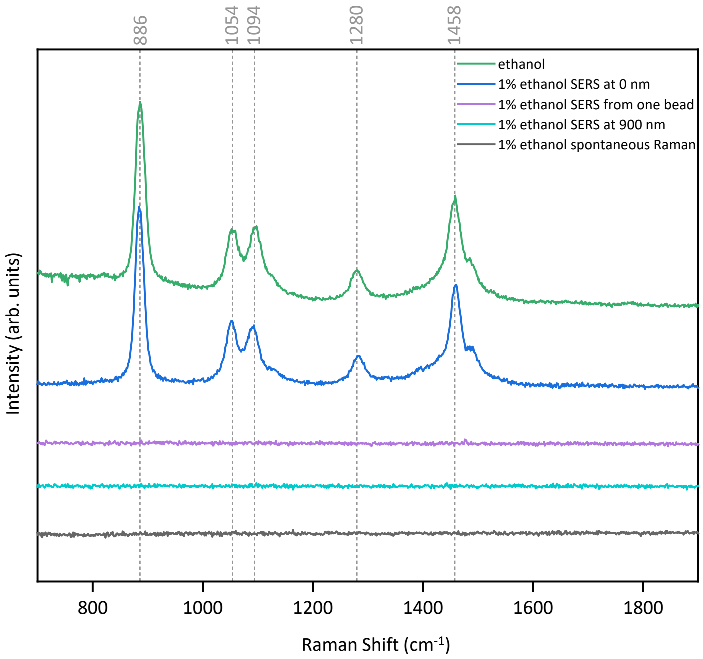

Supplementary Figure 5. The comparison of the spontaneous Raman spectra and the SERS spectra of ethanol solutions. The SERS spectra of 1% ethanol at 0 nm nanogap (1 s, blue) was in good agreement with the spontaneous Raman spectra of pure ethanol (10 s, green). No obvious peaks were observed in both SERS spectra of 1% ethanol when the Raman excitation spot was illuminated on one bead (1 s, purple) or two beads were separated away by 900 nm (1 s, cyan), which were similar to the blank spontaneous Raman spectra of 1% ethanol (10 s, black), indicating no intra-bead hotspots. All the presented spectra were recorded directly upon the subtraction of the spontaneous Raman spectrum of 1% ethanol solution as background.

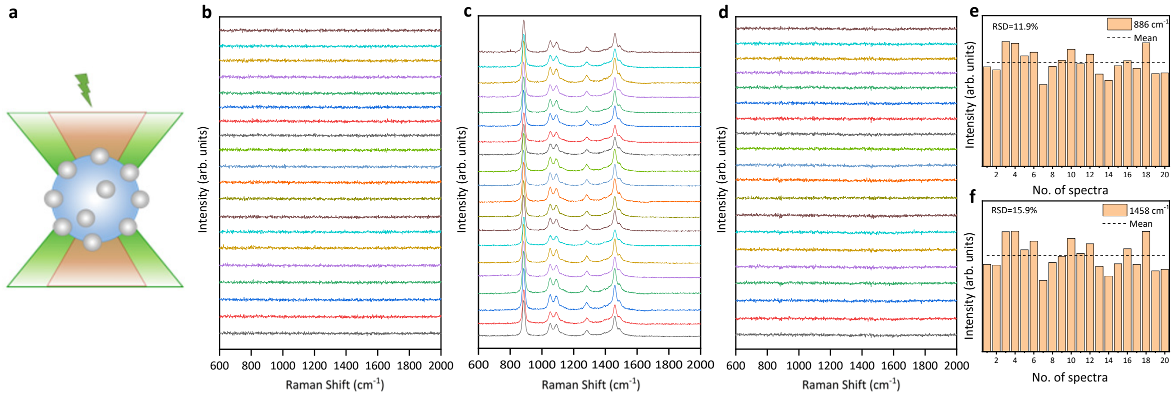

Supplementary Figure 6. Time series SERS spectra of single and approached AgNP-coated beads in 1% ethanol solution. **a**: Schematic diagram of one AgNP-coated bead trapped under Raman excitation spot, where the sizes of the AgNP-coated bead and that of the Raman excitation spot were comparable at around 1 µm and the tapped AgNP-coated bead was in rapid rotation. **b**: Time series SERS spectra of one AgNP-coated bead trapped at the Raman excitation spot in 1% ethanol solution. **c**: Time series SERS spectra of two AgNP-coated beads approached at 0 nm under the Raman excitation spot in 1% ethanol solution. **d**: Time series SERS spectra of one AgNP-coated bead trapped at the Raman excitation spot in 1% ethanol solution when the other bead was moved away after the creation of SERS active interparticle hotspot in **c**. The time series SERS spectra in **b**, **c**, **d** were recorded in continuous 20 s with the acquisition time of 1 s per spectrum. The blank spectra in **b** and **d** indicated

that no active intra-bead hotspot was observed. **e** and **f**: Histogram of peaks intensities of ethanol at 886 cm^{-1} (mean=34497.40 in arb. units, RSD=11.9%) and 1458 cm^{-1} (mean=19137.55 in arb. units, RSD=15.9%) for the 20 SERS spectra in **d**, indicating the stability of enhancement within the created hotspot < 16%.

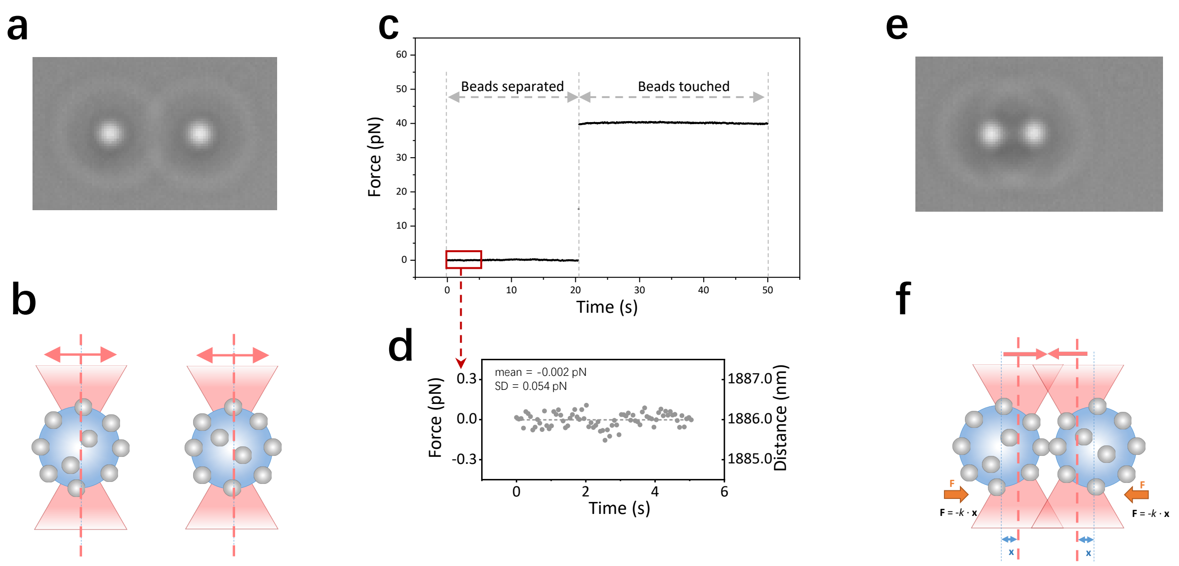

Supplementary Figure 7. **a** and **e**: The real-time camera images of two AgNP-coated beads separated and touched, respectively. **b** and **f**: Illustrations of the force and distance measurements on the two AgNP-coated beads trapped by optical tweezers when two beads are separated and touched. The magnitude of force with respect to the displacement of the bead from the center of the trap follows Hooke's law, $\mathbf{F} = -\mathrm{k} \cdot \mathbf{x}$, where k is the trap stiffness and $\mathbf{x}$ is the bead displacement detected by a position sensitive detector (PSD). The distance between two trapped beads is measured by the camera-based image tracking and the trap position corrected by the bead displacement. **c**: Time traces of the detected force when two AgNP-coated beads were separated and touched. When two trapped AgNP-coated beads were pushed at the bead distance of 0 nm, the force increased sharply from 0 pN to 40 pN, indicating the contact of two AgNP-coated beads to ensure the stability of the interparticle hotspot at the maximum SERS enhancement. **d**: The statistical analysis of the data fluctuation in the first 5 s, giving mean = -0.002 pN and SD < 0.1 pN as the force resolution (SD < 0.3 nm as the spatial resolution at the trap stiffness k = 0.3 pN/nm).

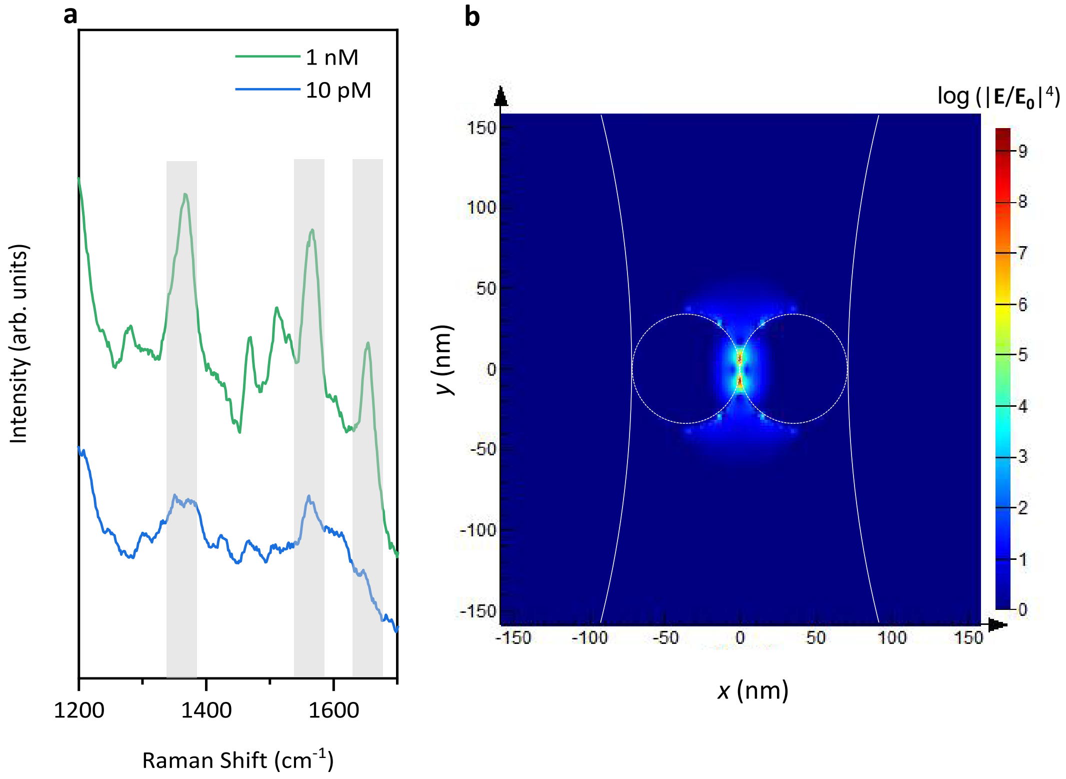

Supplementary Figure 8. SERS measurements and theoretical simulation for the two AgNP-coated beads trapped at 0 nm. **a**: SERS spectra of 1 nM (green) and 10 pM (blue) Rhodamine B with 30 s acquisition time. **b**: FDTD simulation of E-field distribution ($|\mathbf{E}/\mathbf{E_0}|^4$) in logarithm scale, where **E** is the amplified local field and $\mathbf{E_0}$ is the incident field. Dashed circles and solid circles represented the Ag nanoparticles and the silica beads, respectively. Considering the approximation that electromagnetic enhancement factor (EF) can be expressed as $|\mathbf{E}/\mathbf{E_0}|^4$, where **E** is the amplified local field and $\mathbf{E_0}$ is the incident field[1], the maximum EF can reach as high as 10^9 as indicated by the presence of the dark-red spots.

Supplementary Note 1. SERS measurements of Rhodamine B at ultra-low concentration

AgNP-coated beads were incubated in ultra-dilute Rhodamine B solution (10^{-9} M and 10^{-11} M, respectively) for 3 h. After the surface absorption of Rhodamine B, two AgNP-coated beads were trapped and approached at 0 nm under the excitation power of 25 mW to achieve the maximum enhancement for the SERS measurements. In Supplementary Figure 8, the characteristic peaks of Rhodamine B (aromatic C-C stretching at ~1360 cm^{-1}, ~1565 cm^{-1}, and ~1650 cm^{-1}) are observed at 10^{-9} M and even at 10^{-11} M and they are consistent with those at 10^{-9} M, indicating the sensitivity of our SERS platform up to the single-molecule level[2].

Supplementary Note 2. 3D-FDTD Simulation

To better understand the intensity and distribution of electric field in the vicinity of Raman excitation spot, three-dimensional finite-difference time-domain (3D-FDTD) simulation was carried out using FDTD SOLUTIONS provided by Lumerical Solutions, Inc. A simplified model consisting of two Ag nanoparticle (70 nm) coated-silica beads (R=0.63 μm) was constructed. The gap between two AgNP-coated beads was set to 0 nm. The dielectric properties of Ag and SiO_2 were taken from Johnson&Christy database and Palik database, respectively. The refractive index of background fluid was set as 1.33. A 532 nm plane wave propagating along z-axis with polarization parallel to two AgNP-coated beads was employed as excitation source. Perfectly matched layer (PML) boundary condition

was used and the mesh size in the nanogap was set as 0.5 nm to increase the accuracy of simulation. An overall mesh setting with mesh accuracy of 5 was applied for the rest region. Considering the approximation that electromagnetic enhancement factor (EF) can be expressed as $|\mathbf{E}/\mathbf{E_0}|^4$, where **E** is the amplified local field and $\mathbf{E_0}$ is the incident field[1], the maximum EF can reach as high as 10^9 as indicated by the presence of dark-red spots in Supplementary Figure 8b, which is sufficient to empower the single-molecule level detection[3].

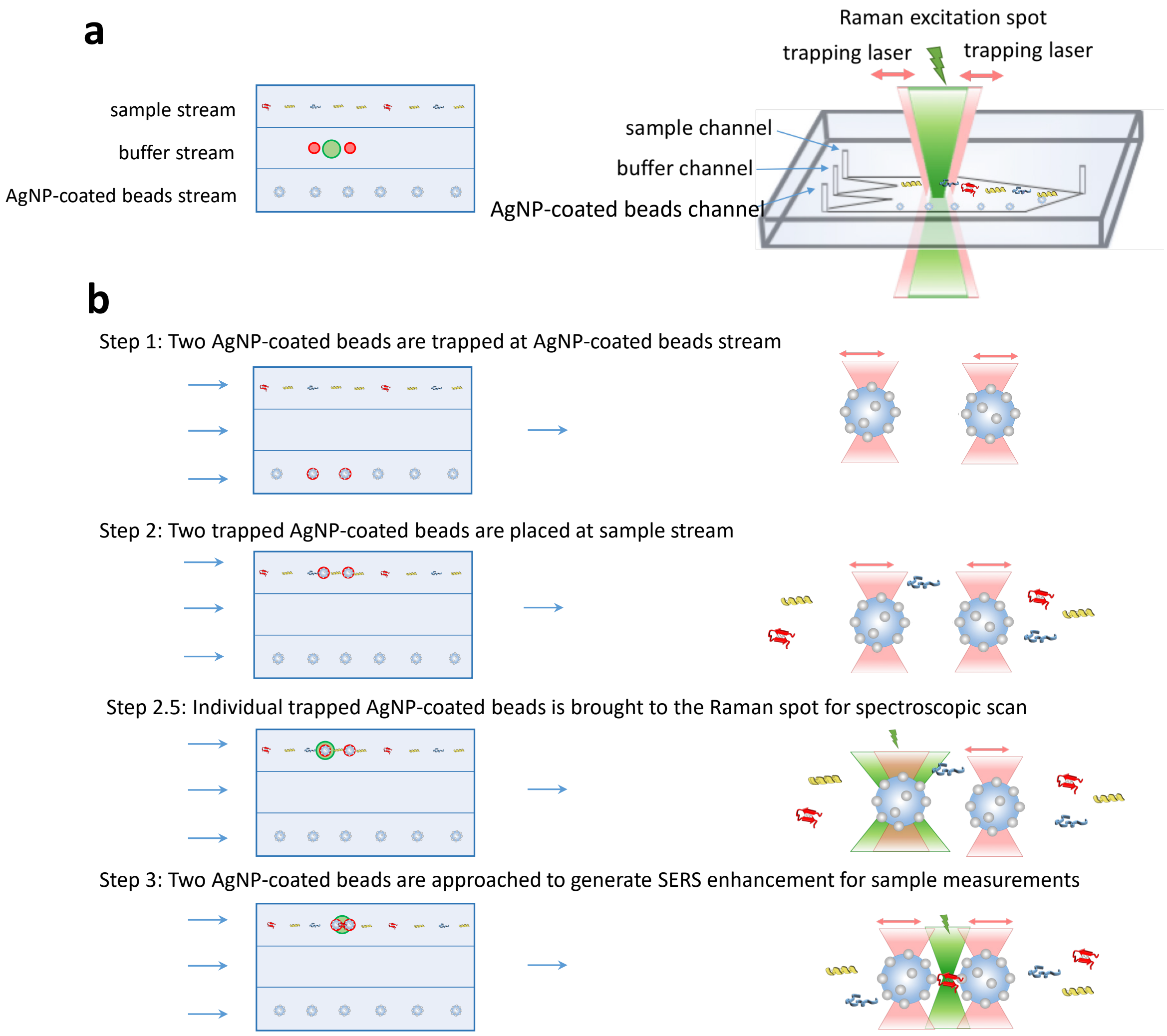

Supplementary Figure 9. **a**: Illustration of the microfluidic flow cell with three adjacent laminar fluidic streams from sample channel, buffer channel, and AgNP-coated bead channel. **b**: Illustration of the detection procedure: Step 1: Two AgNP-coated beads are trapped at AgNP-coated beads stream. Step 2: Two trapped AgNP-coated beads are placed at sample stream. Step 2.5: Individual trapped AgNP-coated beads is brought to the Raman spot for spectroscopic scan. Step 3: Two AgNP-coated beads are approached to generate SERS enhancement for sample measurements.

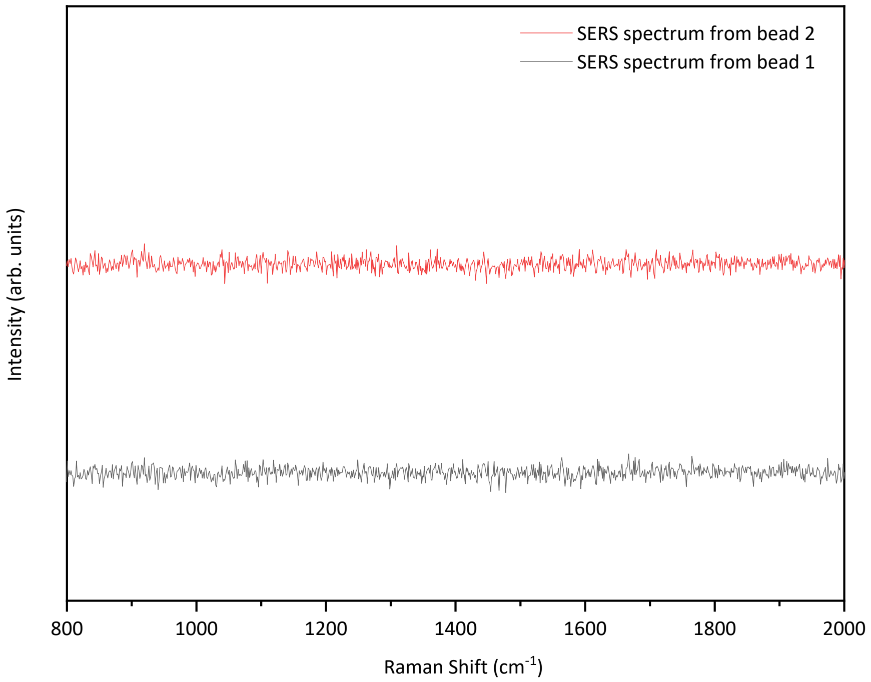

Supplementary Figure 10. SERS spectra of individual trapped AgNP-coated beads at the Raman excitation spot in the sample stream of 100 nM hemoglobin. The blank spectra confirmed neither intra-bead hotspots were formed nor hemoglobin molecules were attached on the surface of beads. The reducing agent trisodium citrate was completely removed to avoid interference.

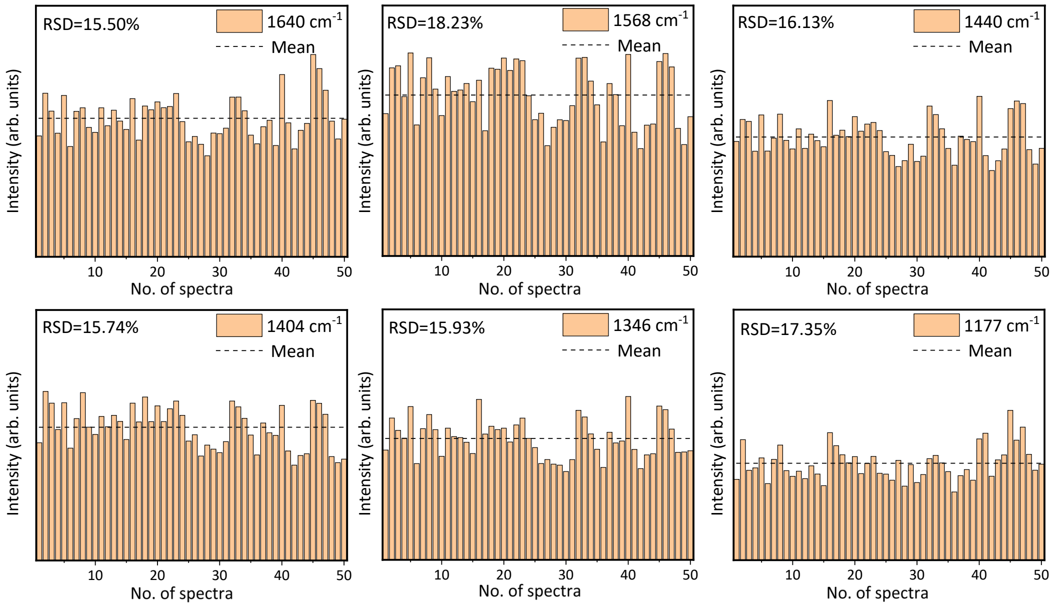

Supplementary Figure 11. The histogram of the intensities of the hemoglobin characteristic peaks at 1640 cm^{-1} (mean=4413.01 in arb. units, RSD=15.50%), 1568 cm^{-1} (mean=5139.80 in arb. units, RSD=18.23%), 1440 cm^{-1} (mean=3811.98 in arb. units, RSD=16.13%), 1404 cm^{-1} (mean=4256.20 in arb. units, RSD=15.74%), 1346 cm^{-1} (mean=3876.91 in arb. units, RSD=15.93%) and 1177 cm^{-1} (mean=3095.36 in arb. units, RSD=17.35%) across the 50 SERS spectra in Fig.3 **d**, respectively. All the RSDs fall within a range of 18.5%, demonstrating reproducibility and stability of our SERS platform.

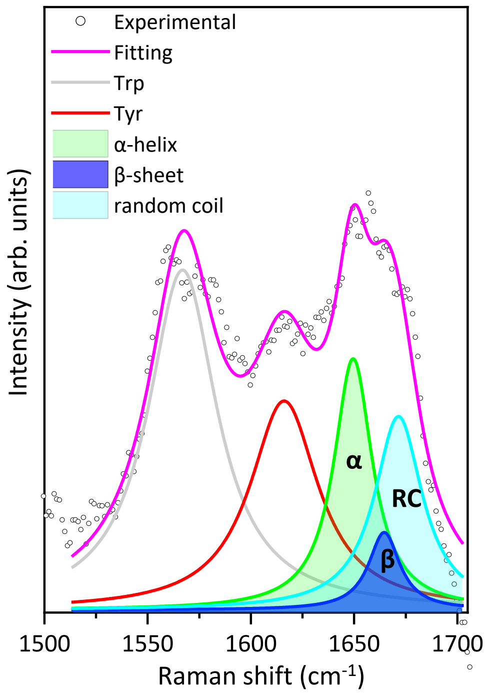

Supplementary Figure 12. Deconvolution of SERS spectrum of 1 μM lysozyme in amide I region. The composition of different secondary structure, such as α-helix (45.2%), β-sheet (11.3%) and random coil (43.5%), is consistent with the previous investigations[4,5].

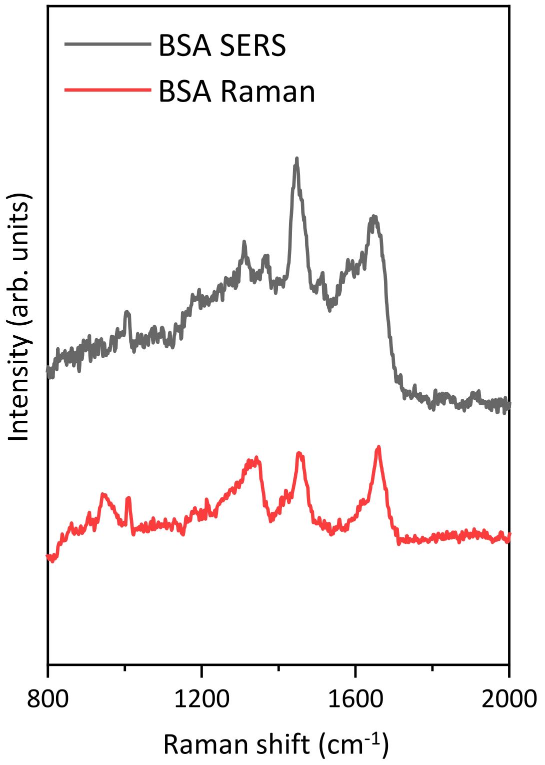

Supplementary Figure 13. SERS and Raman signal of BSA by AgNP-coated beads. SERS signal of 1 μM BSA shows consistent peak position with Raman signal of 1 mM BSA.

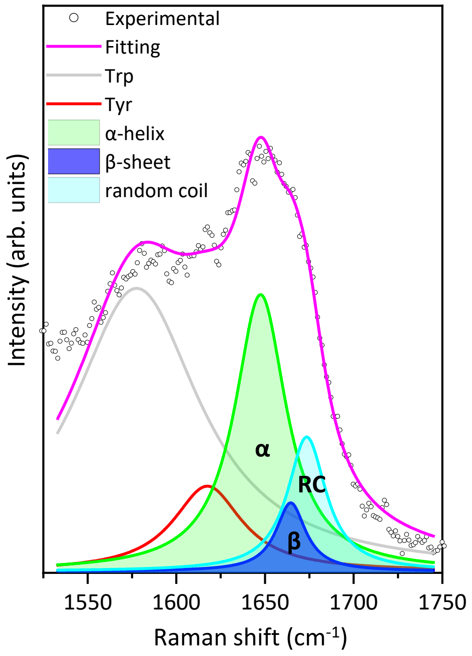

Supplementary Figure 14. Deconvolution of 1 μM BSA SERS signal in amide I region. The composition of different secondary structure, such as α-helix (66.5%), β-sheet (9.5%) and random coil (24.1%), is consistent with the previous studies[6].

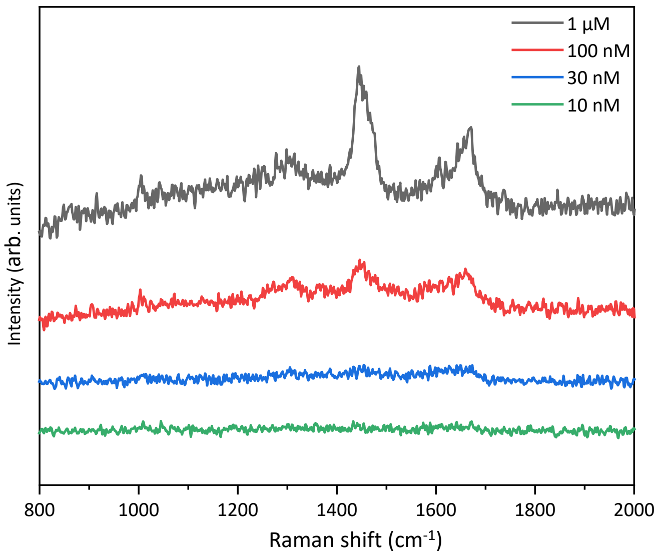

Supplementary Figure 15. SERS spectra of alpha-synuclein at 1 μM, 100 nM, 30 nM and 10 nM obtained at the 20 nm bead distance and the 10% laser power with 1 s acquisition time on our SERS platform, showing the minimum visible concentration of alpha-synuclein aqueous solution is at around 100 nM under the current SERS experimental condition.

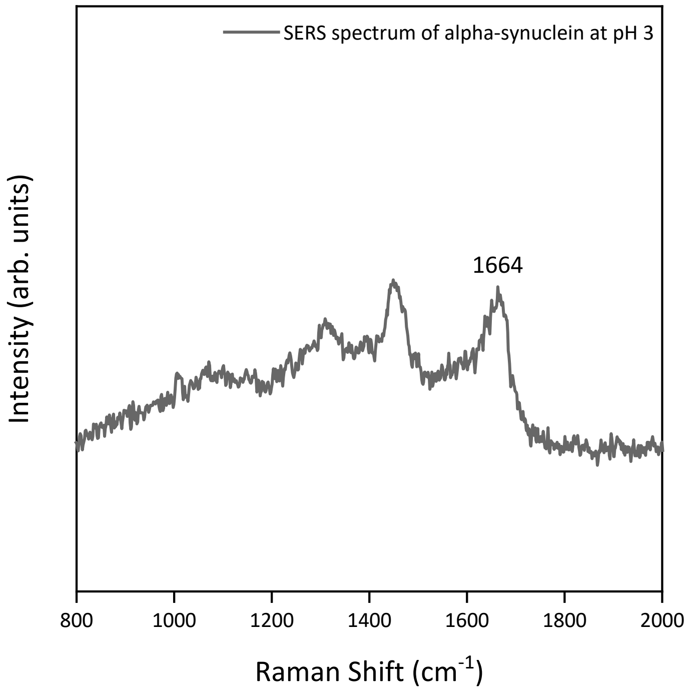

Supplementary Figure 16. SERS spectrum of 1 μM alpha-synuclein at pH 3 with 1 s acquisition time, showing β-sheet conformation.

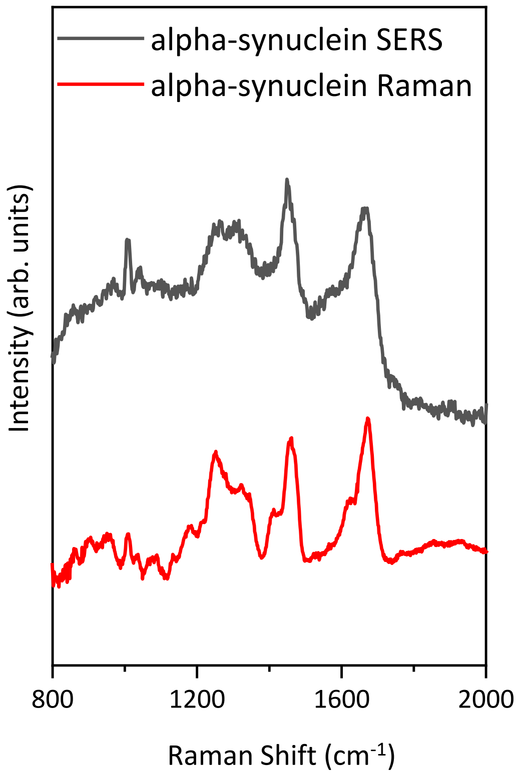

Supplementary Figure 17. SERS (250 μM) and spontaneous Raman (2 mM) spectra of alpha-synuclein.

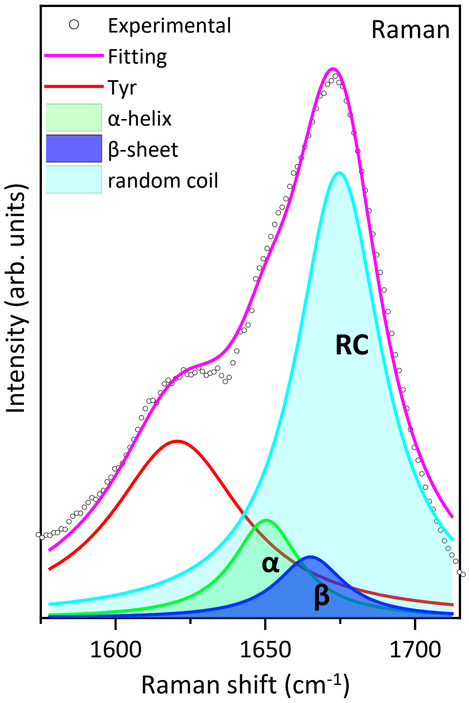

Supplementary Figure 18. Deconvolution of the spontaneous Raman spectrum of alpha-synuclein in amide I region. The composition of different secondary structure from Raman (13.3% α-helix, 8.4% β-sheet and 78.3% random coil) is consistent with the previous study[7,8].

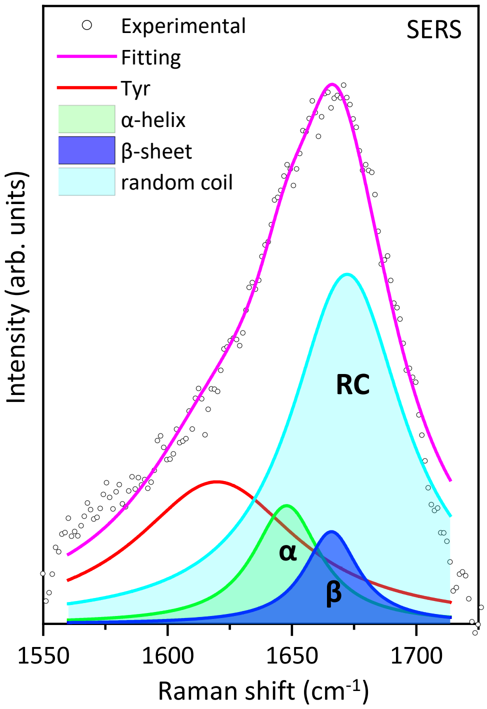

Supplementary Figure 19. Deconvolution of SERS spectrum of alpha-synuclein in amide I region. The composition of different secondary structure from SERS (15.1% α-helix, 9.5% β-sheet and 75.4% random coil) is similar to that from Raman.

Supplementary Note 3. Amide I band deconvolution

To reveal the secondary structural components of lysozyme, BSA and alpha-synuclein, the broad amide I bands were deconvoluted based on the Levenberg–Marquardt algorithm. Lorentz profiles were employed during the deconvolution procedure. Three amide I bands were centered at 1648-1656 cm^{-1}, 1662-1665 cm^{-1}, 1668-1680 cm^{-1} and attributed to α-helix, β-sheet and random coil, respectively. Additional peaks at ~1620 cm^{-1} and ~1560 cm^{-1} associated with aromatic side chains were included during the deconvolution procedure. All the spectra were smoothed by Savitsky–Golay filter prior to perform deconvolution. The integral areas of the deconvoluted bands were used to estimate the ratio of these three secondary structural components.

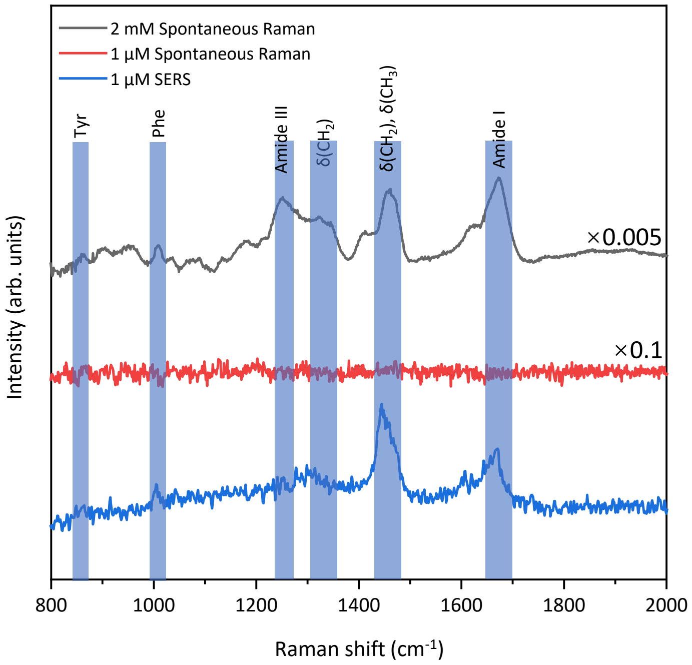

Supplementary Figure 20. The comparison of the SERS spectrum of 1 μM alpha-synuclein solution (1 s acquisition time) and the spontaneous Raman spectra of 1 μM and 2 mM alpha-synuclein solution (10 min acquisition time).

Supplementary Table 1. The vibrational bands assignment of hemoglobin.

Raman Shift (cm^{-1})	Assignment*
755	ν_{15}, ν(pyr breathing)
1006	ν_{45}, $\nu(C_\alpha\text{-}C_1)_{\mathbf{asym}}$
1089	$\delta(=CH_2)_{\mathbf{asym}}$
1128	ν_{22}, $\nu(C_\alpha\text{-}N)$
1177	ν_{30}, $\nu(\text{pyr half-ring})_{\mathbf{asym}}$
1232	ν_{13}, $\delta(C_m\text{-}H)$
1311	δ(CH=)
1346 for Fe(II)<br>1376 for Fe(III)	ν_4, $\nu(\text{pyr half-ring})_{\mathbf{sym}}$
1404	ν_{29}, $\nu(\text{pyr quarter-ring})_{\mathbf{sym}}$
1440	$\delta_s(=CH_2)$
1508 for 6cLS	ν_3, $\nu(C_\alpha\text{-}C_m)_{sym}$
1568 for 6cHS<br>1594 for 6cLS	ν_2, $\nu(C_\beta\text{-}C_\beta)$
1640	ν_{10}, $\nu(C_\alpha\text{-}C_m)_{asym}$

*Assignments are based on the studies by Hu et al.[9], Kalaivani et al.[10], Wood et al.[11], Casella et al.[12], and Mizutani[13].

Supplementary Table 2. The vibrational bands assignment of alpha-synuclein.

Raman Shift (cm^{-1})	Assignment*
860	Tyr
905	*v*(C-C)
950	*v*(C-C)
1006	Phe
1090	*v*(C-C), *v*(C-N), *v*(C-O)
1134	*v*(C-C)
1177	Tyr, Phe
1211	Tyr
1230-1240 for β-sheet	
1240-1250 for Random coil	Amide III
1270-1300 for α-helix	
1322	CH_2 deformation
1346	CH_2 deformation
1450	CH_2, CH_3 deformation
1604	Phe
1620	Tyr
1653 for α-helix	
1664 for β-sheet	Amide I
1671 for Random coil	

* Assignments are based on the study by Maiti et al.[14].

References:

1. Lee, H. M., Jin, S. M., Kim, H. M. & Suh, Y. D. Single-molecule surface-enhanced Raman spectroscopy: A perspective on the current status. *Phys. Chem. Chem. Phys.* **15**, 5276–5287 (2013).
2. Xu, H., Bjerneld, E. J., Käll, M. & Börjesson, L. Spectroscopy of single hemoglobin molecules by surface enhanced raman scattering. *Phys. Rev. Lett.* **83**, 4357–4360 (1999).
3. Lim, D. K., Jeon, K. S., Kim, H. M., Nam, J. M. & Suh, Y. D. Nanogap-engineerable raman-active nanodumbbells for single-molecule detection. *Nat. Mater.* **9**, 60–67 (2010).
4. Lippert, J. L., Tyminski, D. & Desmeules, P. J. Determination of the Secondary Structure of Proteins by Laser Raman Spectroscopy. *J. Am. Chem. Soc.* **98**, 7075–7080 (1976).
5. Phillips, D. C. The hen egg-white lysozyme molecule. *Proc. Natl. Acad. Sci.* **57**, 483–495 (1967).
6. Peters Jr, T. *All about albumin: biochemistry, genetics, and medical applications*. (Academic Press, 1995).
7. Davidson, W. S., Jonas, A., Clayton, D. F. & George, J. M. Stabilization of α-Synuclein secondary structure upon binding to synthetic membranes. *J. Biol. Chem.* **273**, 9443–9449 (1998).
8. Zand, Z. *et al.* Cerium oxide NPs mitigate the amyloid formation of α-synuclein and associated cytotoxicity. *Int. J. Nanomedicine* **14**, 6989–7000 (2019).
9. Hu, S., Smith, K. M. & Spiro, T. G. Assignment of protoheme Resonance Raman spectrum by heme labeling in myoglobin. *J. Am. Chem. Soc.* **118**, 12638–12646 (1996).
10. Kalaivani, G. *et al.* Plasmon-tuned silver colloids for SERRS analysis of methemoglobin with preserved nativity. *Langmuir* **28**, 14357–14363 (2012).
11. Wood, B. R., Kochan, K. & Marzec, K. M. *Resonance Raman spectroscopy of hemoglobin in red blood cells*. *Vibrational Spectroscopy in Protein Research* (INC, 2020). doi:10.1016/b978-0-12-818610-7.00013-x.
12. Casella, M. *et al.* Raman and SERS recognition of β-carotene and haemoglobin fingerprints in human whole blood. *Spectrochim. Acta - Part A Mol. Biomol. Spectrosc.* **79**, 915–919 (2011).
13. Mizutani, Y. *Dynamics and allostery of human hemoglobin as elucidated by time-resolved resonance Raman spectroscopy*. *Vibrational Spectroscopy in Protein Research* (INC, 2020). doi:10.1016/b978-0-12-818610-7.00016-5.
14. Maiti, N. C., Apetri, M. M., Zagorski, M. G., Carey, P. R. & Anderson, V. E. Raman Spectroscopic Characterization of Secondary Structure in Natively Unfolded Proteins: α-Synuclein. *J. Am. Chem. Soc.* **126**, 2399–2408 (2004).
