# Supplementary material for: Optical tweezers-controlled hotspot for sensitive and reproducible surface-enhanced Raman spectroscopy characterization of native protein structures
Source: Nat Commun. 2021 Feb 26;12:1292. doi: 10.1038/s41467-021-21543-3 (PMC7910584; doi:10.1038/s41467-021-21543-3)
Supplement: Supplementary file 2 — Reporting Summary [file 41467_2021_21543_MOESM2_ESM.pdf]

nature research

Corresponding author(s): Dr. Jinqing Huang

Last updated by author(s): Jan 17, 2021

Reporting Summary

Nature Research wishes to improve the reproducibility of the work that we publish. This form provides structure for consistency and transparency in reporting. For further information on Nature Research policies, see our Editorial Policies and the Editorial Policy Checklist.

Statistics

For all statistical analyses, confirm that the following items are present in the figure legend, table legend, main text, or Methods section.

n/a	Confirmed	
☐	☒	The exact sample size (*n*) for each experimental group/condition, given as a discrete number and unit of measurement
☐	☒	A statement on whether measurements were taken from distinct samples or whether the same sample was measured repeatedly
☒	☐	The statistical test(s) used AND whether they are one- or two-sided<br>*Only common tests should be described solely by name; describe more complex techniques in the Methods section.*
☒	☐	A description of all covariates tested
☒	☐	A description of any assumptions or corrections, such as tests of normality and adjustment for multiple comparisons
☐	☒	A full description of the statistical parameters including central tendency (e.g. means) or other basic estimates (e.g. regression coefficient) AND variation (e.g. standard deviation) or associated estimates of uncertainty (e.g. confidence intervals)
☒	☐	For null hypothesis testing, the test statistic (e.g. *F*, *t*, *r*) with confidence intervals, effect sizes, degrees of freedom and *P* value noted<br>*Give P values as exact values whenever suitable.*
☒	☐	For Bayesian analysis, information on the choice of priors and Markov chain Monte Carlo settings
☒	☐	For hierarchical and complex designs, identification of the appropriate level for tests and full reporting of outcomes
☒	☐	Estimates of effect sizes (e.g. Cohen's *d*, Pearson's *r*), indicating how they were calculated

Our web collection on statistics for biologists contains articles on many of the points above.

Software and code

Policy information about availability of computer code

Data collection	Spectral data was collected using Winspec for the spectrometer (Teledyne Princeton Instruments, USA).<br>Bead distance and force data was collected using Bluelake for the optical tweezers microscope (Lumicks B. V., Netherlands).
Data analysis	Data was analyzed and plotted using OriginPro 2021 (v9.8). The custom MATLAB (2019b) code was used to simulate the Brownian motion of trapped particles. The three-dimensional finite-difference time-domain (3D-FDTD) simulation was conducted using FDTD SOLUTIONS, 2018a (8.19.1584) provided by Lumerical Solutions, Inc. All codes with instructions are shared on-line in DataSpace at: https://doi.org/10.14711/dataset/DXO9VR. The links and access codes are also provided in the code availability section of the manuscript.

For manuscripts utilizing custom algorithms or software that are central to the research but not yet described in published literature, software must be made available to editors and reviewers. We strongly encourage code deposition in a community repository (e.g. GitHub). See the Nature Research guidelines for submitting code & software for further information.

Data

Policy information about availability of data

All manuscripts must include a data availability statement. This statement should provide the following information, where applicable:

- Accession codes, unique identifiers, or web links for publicly available datasets
- A list of figures that have associated raw data
- A description of any restrictions on data availability

Source data are provided with this paper, underlying Fig. 2, 3, 4, 5 and supplementary figures. The raw data that support the findings of this study are available in DataSpace at: https://doi.org/10.14711/dataset/DXO9VR.

Field-specific reporting

Please select the one below that is the best fit for your research. If you are not sure, read the appropriate sections before making your selection.

☒ Life sciences ☐ Behavioural & social sciences ☐ Ecological, evolutionary & environmental sciences

For a reference copy of the document with all sections, see nature.com/documents/nr-reporting-summary-flat.pdf

Life sciences study design

All studies must disclose on these points even when the disclosure is negative.

Sample size	No sample size calculation was performed. The parallel spectroscopic characterizations were performed on four individual proteins (hemoglobin, lysozyme, bovine serum albumin, and alpha-synuclein) in dilute aqueous solution, based on the standards of the field. We have conducted parallel spectroscopic measurements on 1 uM alpha-synuclein for 200 times, 1 uM lysozyme for 50 times, and 100 nM hemoglobin for 50 times. We have evaluated the data across more than 5 different batches of the protein samples. The protein samples are very stable from batch to batch and the spectral results are highly reproducible. Hence, these parallel spectroscopic measurements could provide sufficient number of spectra as the effective sample size for the protein structural characterizations.
Data exclusions	No data was excluded.
Replication	We have conducted parallel spectroscopic measurements on 1 uM alpha-synuclein for 200 times, 1 uM lysozyme for 50 times, and 100 nM hemoglobin for 50 times. We have evaluated the data across more than 5 different batches of the protein samples. All replications were consistent and successful.
Randomization	Randomization was not used since there were no experimental groups.
Blinding	Blinding was not used since there were no experimental groups.

Reporting for specific materials, systems and methods

We require information from authors about some types of materials, experimental systems and methods used in many studies. Here, indicate whether each material, system or method listed is relevant to your study. If you are not sure if a list item applies to your research, read the appropriate section before selecting a response.

Materials & experimental systems

n/a	Involved in the study	
☒	☐	Antibodies
☒	☐	Eukaryotic cell lines
☒	☐	Palaeontology and archaeology
☒	☐	Animals and other organisms
☒	☐	Human research participants
☒	☐	Clinical data
☒	☐	Dual use research of concern

Methods

n/a	Involved in the study	
☒	☐	ChIP-seq
☒	☐	Flow cytometry
☒	☐	MRI-based neuroimaging
